# Disseminated tuberculosis after pregnancy progressed to paradoxical response to the treatment: report of two cases

**DOI:** 10.1186/s12879-016-1624-x

**Published:** 2016-06-13

**Authors:** Tsutomu Shinohara, Kozo Kagawa, Yoshio Okano, Toru Sawada, Tooru Kobayashi, Masaya Takikawa, Yoshihito Iwahara, Fumitaka Ogushi

**Affiliations:** Department of Clinical Investigation, National Hospital Organization Kochi Hospital, 1-2-25 Asakuranishimachi, Kochi, 780-8077 Japan; Division of Pulmonary Medicine, National Hospital Organization Kochi Hospital, 1-2-25 Asakuranishimachi, Kochi, 780-8077 Japan; Division of Thoracic Surgery, National Hospital Organization Kochi Hospital, 1-2-25 Asakuranishimachi, Kochi, 780-8077 Japan; Division of Orthopaedic Surgery, National Hospital Organization Kochi Hospital, 1-2-25 Asakuranishimachi, Kochi, 780-8077 Japan; Division of Obstetrics and Gynecology, National Hospital Organization Kochi Hospital, 1-2-25 Asakuranishimachi, Kochi, 780-8077 Japan; Division of Hematology, National Hospital Organization Kochi Hospital, 1-2-25 Asakuranishimachi, Kochi, 780-8077 Japan

**Keywords:** Tuberculosis, Pregnancy, Paradoxical response

## Abstract

**Background:**

Early postpartum women are more likely to develop tuberculosis than nonpregnant women mainly due to immune reconstitution after delivery. Paradoxical response (PR) during antituberculosis treatment also arises via recovery from immunosuppression. However, no study focused on PR during antituberculosis treatment in a postpartum patient has been reported.

**Case presentation:**

We present two sequential cases (Patient 1: 26-year-old; Patient 2: 29-year-old) of postpartum tuberculosis with pulmonary and extrapulmonary lesions (Patient 1: peritonitis; Patient 2: psoas abscess secondary to spondylitis). Both cases progressed to PR (worsening of pre-existing lung infiltrations (Patients 1, 2) and new contralateral effusion (Patient 2)) in a relatively short time after initiation of treatment (Patient 1: 1 week; Patient 2: 3 weeks), suggesting that immune modulations during pregnancy and delivery may contribute to the pathogenesis of both disseminated tuberculosis and its PR. The pulmonary lesions and effusion of both cases gradually improved without change of chemotherapy regimen.

**Conclusion:**

Physicians should recognize PR in tuberculosis patients with postpartum and then evaluate treatment efficacy.

## Background

Pregnancy is a relatively immunosuppressive state to escape maternal anti-fetus rejection. Soon after delivery, T-helper 1 suppression was shown to reverse rapidly, and cellular immune reconstitution is thought to be a major cause of postpartum exacerbation of otherwise quiescent or latent infections [1]. Representative infectious etiologies of exacerbation after pregnancy include *Cryptococcus neoformans*, hepatitis and Herpes viruses, and *Mycobacterium tuberculosis* [1, 2]. Early (within 6 months) postpartum women are twice as likely to develop tuberculosis as nonpregnant women [2]. Although management of tuberculosis in pregnant and postpartum female has been discussed [3, 4], no study focused on paradoxical response (PR) during antituberculosis treatment in a postpartum patient has been reported.

PR during antituberculosis treatment is defined as the transient clinical or radiological worsening or new formation of tuberculous lesions that occur after initiation of appropriate chemotherapy, and is not due to treatment failure or the presence of another diagnosis. It is thought that the hypersensitivity response to mycobacterial antigens following immune reconstitution is the basis of PR pathogenesis [5, 6]. In this report, we present two sequential cases of postpartum tuberculosis with pulmonary and extrapulmonary lesions, both of which progressed to PR to the treatment, suggesting that immune modulations during pregnancy and delivery may contribute to the pathogenesis of both disseminated tuberculosis and its PR.

## Case presentation

### Patient 1

**Fig. 1:**
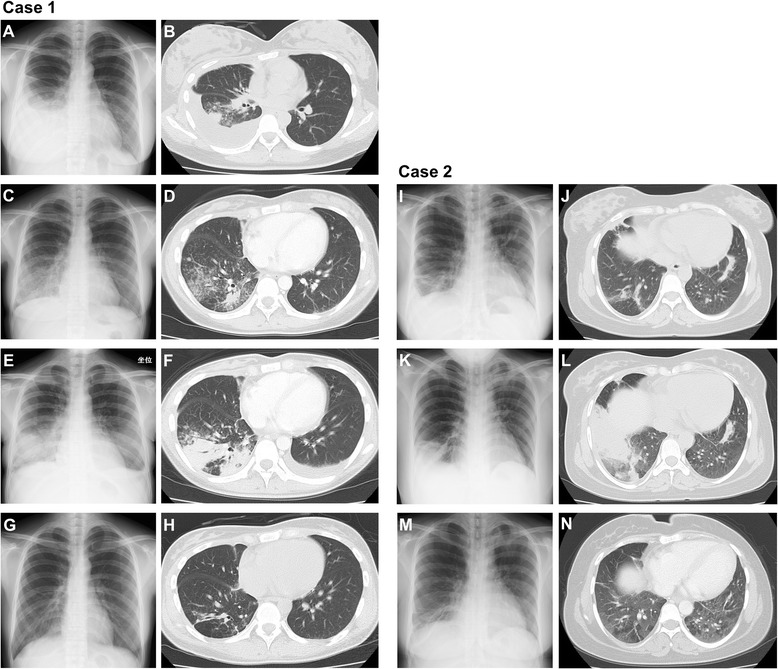
Time course of chest X-ray and CT findings of case 1 (**a**–**h**) and case 2 (**i**–**n**). **a** and **b**, Right pleural effusion and homonymous lung infiltration 1 month after delivery. **c** and **d**, Residual lung infiltration without effusion 4 months after delivery. **e** and **f**, Worsening of pre-existing lung infiltrations with new contralateral effusion 4 weeks after initiation of the antituberculosis treatment. **g** and **h**, Disappearance of effusion and improvement of lung infiltrations after a course of chemotherapy (2HREZ/7HR). **i** and **j**, Multiple infiltrative shadows in lungs 6 months after delivery. **k** and **l**, Worsening of infiltrations in the right lung 10 weeks after initiation of the antituberculosis treatment. **m** and **n**, Improvement in lung infiltrations after a course of chemotherapy (2HREZ/7HR)

A 26-year-old female with an uneventful antenatal period delivered a full-term baby by normal vaginal delivery. She developed persistent fever 1 month after delivery. Chest X-ray and computed tomography (CT) showed right pleural effusion and homonymous lung infiltration (Fig. 1a and b), which was not observed before delivery. Patient was initially treated as a case of bacterial pleuropneumonia with parenteral antibiotics during 3 weeks of hospitalization with decline of fever; pleural effusion decreased gradually for 3 months.

**Fig. 2:**
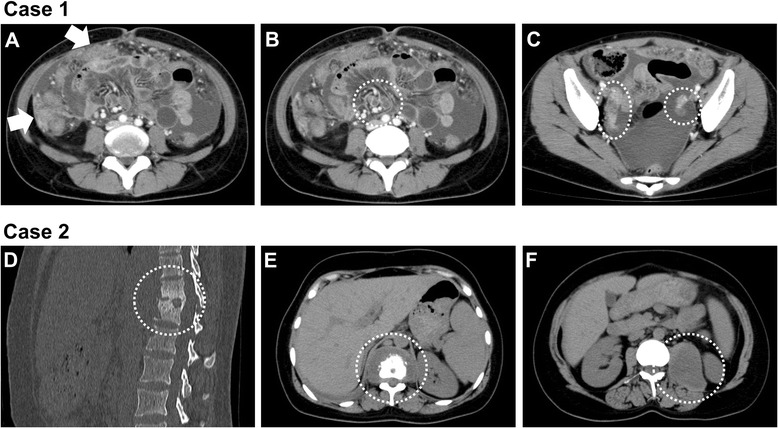
Abdominal CT findings of case 1 (**a**–**c**) and case 2 (**d**–**f**) at diagnosis. **a**, Moderate ascites and intraabdominal mass lesions (*arrows*). **b**, intraabdominal lymph nodes swelling (*in the circle*). **c**, bilateral adnexal masses (*in the circle*). **d** and **e**, Osteosclerotic lesion of Th11-Th12 vertebra with surrounding abscess (*in the circle*). **f**, A large low attenuation mass in the left psoas muscle (*in the circle*)

However, fever recurred 4 months after the delivery with gradually worsening abdominal distention, and she was admitted to our department. Chest X-ray and CT showed residual lung infiltrations without cavity and effusion (Fig. 1c and d). Abdominal CT revealed moderate ascites, intraabdominal mass lesions and lymph nodes swelling, and bilateral adnexal masses (Fig. 2a, b and c). Diagnostic puncture yielded clear yellow fluid, which was consistent with exudates. The sputum and ascites acid-fast bacillus smear were negative, and both cultures isolated no common bacteria. However, the QuantiFERON-TB third generation (QFT-3G) test was positive, and the diagnosis of pulmonary and peritoneal tuberculosis was established by the detection of *M. tuberculosis* DNA in sputum and ascites utilizing PCR. Although the CA125 serum level was high (247 IU/ml), ascitic cytology did not reveal malignant cells. Further serology tests were negative for antinuclear antibody and HIV-Ab. Pulmonary and peritoneal tuberculosis were later confirmed by acid-fast bacilli cultures.

The patient was started on quadruple therapy (isoniazid, rifampin, ethambutol and pyrazinamide: HREZ). One week after the chemotherapy was started, worsening of the pre-existing lung infiltrations with new contralateral effusion was noted, and continued for 3 weeks (Fig. 1e and f). Improvement of abdominal findings was not observed in this period. However, the chemotherapy regimen was not altered, since this strain of *M. tuberculosis* was sensitive to all principal antituberculous drugs. Then, the lung infiltrations and effusion were gradually improved by continuation of chemotherapy (Fig. 1g and h). The sputum acid-fast bacilli cultures became negative and ascites disappeared. The CA125 serum level was reduced to the normal range after chemotherapy.

### Patient 2

A 29-year-old woman underwent a cesarean section at 36 weeks gestation. Although she began to notice left lumbar backache 2 weeks after the operation, further examination was not carried out. Five months later, she was admitted to our hospital due to prominent left lumbar backache with newly appeared productive cough. An abdominal CT revealed spondylitis of the Th11-Th12 vertebra and a large low attenuation mass in the left psoas muscle, at a size of 7 cm × 5 cm × 8 cm (Fig. 2d, e and f). In addition, chest X-ray and CT showed multiple infiltrative shadows in the lungs (Fig. 1i and j). CT-guided percutaneous needle biopsy of the psoas abscess was performed, obtaining a dense, purulent fluid. Acid-fast bacillus staining and PCR tests for M. tuberculosis of the fluid were both positive. No bacteria or fungi were isolated from the same samples. In addition, T-SPOT.TB was also positive. Tuberculous psoas abscess and pulmonary tuberculosis were later confirmed by acid-fast bacilli cultures.

The patient was started on quadruple therapy (HREZ), and a CT-guided percutaneous drainage of the psoas abscess was performed. Three week after the chemotherapy was started, right side chest pain developed. Subsequently, radiological worsening of infiltrations in the right lung were observed, and continued for 7 weeks (Fig. 1k and l). However, the pulmonary infiltrations gradually improved without change of the chemotherapy regimen (Fig. 1m and n). The strain of *M. tuberculosis* was sensitive to all principal antituberculous drugs, and acid-fast bacilli cultures became negative in sputum and needle aspiration specimens of the small residual psoas abscess.

## Discussion

During pregnancy, Th2/Th3 responses, which lead to immunosuppressive and anti-inflammatory responses, are enhanced and the Th1 response, which leads to proinflammatory responses, is suppressed, preventing the maternal immune system from assaulting the fetus. Maternal hormones and placental products play an important role in these immune modulations. Th2 response facilitates the asymptomatic mycobacterial infection and the shift from Th2 towards Th1 response during the postpartum period induces active diseases mostly with extrapulmonary or disseminated lesions from the latent state. Such pathogenesis is considered to be part of immune reconstitution syndrome (IRS) [1, 7], and similarities between IRS and PR in tuberculosis have been reported [8].

The fact that initiation of anti-HIV therapy for patients with AIDS or discontinuation of anti-TNFα therapy for patients with autoimmune disease after initiation of tuberculosis treatment is a representative risk factor for PR suggests that hypersensitivity response to mycobacterial antigens following immune reconstitution is the basis of PR pathogenesis [5, 6]. Increased circulating TNFα (one of the main proinflammatory cytokines) with concomitant clinical deterioration after the initiation of therapy in HIV-negative (HIV^−^) patients with severe tuberculosis has also been reported [9]. PR is significantly more frequent among patients with extrapulmonary or disseminated tuberculosis, as shown in our patients [5, 6]. In addition, cases of paradoxical inflammatory reaction during treatment of cryptococcal meningitis in postpartum females with or without HIV infection were reported [10, 11]. However, to the best of our knowledge, no study focused on PR during antituberculosis treatment in a postpartum patient has been reported except for this manuscript.

PR has been well described among otherwise healthy patients treated for tuberculosis [12], although their risk of PR is lower than that in tuberculosis patients with HIV co-infection [13]. The mechanisms of PR in otherwise healthy patients have been understood as recovery from tuberculosis-induced immunosuppression leading to a local hypersensitive response against massive mycobacterial antigens exposure following antituberculosis treatment. Generally initial improvement on antituberculosis treatment is observed before PR in HIV^−^ patients [12], and the time from starting antituberculosis treatment to PR is longer in HIV^−^ patients as compared to HIV-positive (HIV^+^) patients. Breen et al. reported that the median time from starting antituberculosis treatment to PR was 87 days in HIV^−^ patients. However, in HIV^+^ tuberculosis patients the median time between starting antiretroviral therapy and PR was only 11 days [13]. Since the times from starting tuberculosis treatment to PR were relatively short in our cases (1 week and 3 weeks) compared to typical HIV^−^ patients, PR in a postpartum patient may be attributed to the continuation of a preexisting hypersensitivity response to mycobacterial antigens after initiation of tuberculosis treatment.

The onset of the first patient may be retrospectively considered to be tuberclous pleurisy during the postpartum period, although antibiotics therapy seemed to be successful. The pathogenesis of tuberculous pleurisy is a delayed-type hypersensitivity immunogenic reaction to a few mycobacterial antigens entering the pleural space, and resolution of the pleural effusion usually occurs spontaneously without antituberculous chemotherapy. However, about half of untreated cases of primary tuberculous pleurisy develop into more severe forms of active pulmonary and/or extra-pulmonary tuberculosis [14]. Moreover, a previous report indicated that about 90 % of the patients with postpartum tuberculosis had extra-pulmonary tuberculosis, including pleuritis, peritonitis and CNS infection [7]. Therefore, we speculate that the entire course of the first patient is consistent with the progression of postpartum tuberculosis.

Peritoneal tuberculosis is occasionally accompanied by adnexal lesions, and elevated serum CA125 level may also be found in cases of pelvic inflammation as shown in our case [15]. Therefore the clinical features of peritoneal tuberculosis rather resemble ovarian carcinoma, diagnosis of which usually leads to radical surgery. Actually, a number of patients, who were suspected preoperatively as having ovarian malignancy, proved to be peritoneal tuberculosis by resected specimens [15]. In addition, several cases of abdominal tuberculosis, which deteriorated during antituberculosis treatment as PR, have been reported [12, 16, 17]. For postpartum patients with peritoneal tuberculosis mimicking ovarian cancer, there is the additional consideration of PR during chemotherapy to avoid erroneously judging that deterioration during antituberculosis treatment is due to a coexisting malignancy.

Historically the majority of psoas abscesses were caused by tuberculosis of the vertebra, one of the typical manifestations of extrapulmonary tuberculosis. More recently, psoas abscesses are mainly caused by *Staphylococcus aureus* as a primary abscess [18]. Although several bacterial psoas abscesses have been reported during pregnancy and subsequent to normal vaginal delivery, cesarean section and abortion, tuberculous psoas abscess in obstetrics is extremely rare and only two cases during pregnancy have been reported [19]. Our second case is the first report of postpartum tuberculous psoas abscess, which is secondary to spondylitis. Persistent lower back pain in postpartum women should be investigated with clinical suspicion of a psoas abscess and/or spondylitis. Interestingly, a case of a young man with systemic lymph node tuberculosis which progressed to psoas abscess caused by a PR has been reported [20], suggesting that, as with lower back pain, we need to consider the possibility of psoas abscess even after the start of antituberculosis treatment.

The prognosis of postpartum tuberculosis is relatively poor [1]. Therefore, more clinical and basic studies are needed in order to allow better management of PR in postpartum tuberculosis. Especially, the use of systemic steroids to suppress the hypersensitivity response in postpartum patients should be evaluated, since steroid treatment for PR in HIV^−^ patients is controversial over the owing to the absence of randomized controlled studies.

## Conclusion

In summary, careful observation with systemic examinations on various extrapulmonary lesions is essential for clinical practice in postpartum tuberculous, and recognition of the PR within a relatively short time after the initiation of treatment is necessary to evaluate the effectiveness of the treatment.

## Abbreviations

CT, computed tomography; HIV^−^, HIV-negative; HIV^+^, HIV-positive; HREZ, isoniazid, rifampin, ethambutol and pyrazinamide; IRS, immune reconstitution syndrome; PR, paradoxical response

## References

[1] Singh N, Perfect JR (2007). Immune reconstitution syndrome and exacerbation of infections after pregnancy. Clin Infect Dis.

[2] Zenner D, Kruijshaar ME, Andrews N, Abubakar I (2012). Risk of tuberculosis in pregnancy: a national, primary care-based cohort and self-controlled case series study. Am J Respir Crit Care Med.

[3] Mathad JS, Gupta A (2012). Tuberculosis in pregnant and postpartum women: epidemiology, management, and research gaps. Clin Infect Dis.

[4] Ormerod P (2001). Tuberculosis in pregnancy and the puerperium. Thorax.

[5] Narita M, Ashkin D, Hollender ES, Pitchenik AE (1998). Paradoxical worsening of tuberculosis following antiretroviral therapy in patients with AIDS. Am J Respir Crit Care Med.

[6] Garcia Vidal C, Rodríguez Fernández S, Martínez Lacasa J, Salavert M, Vidal R, Rodríguez Carballeira M (2005). Paradoxical response to antituberculous therapy in infliximab-treated patients with disseminated tuberculosis. Clin Infect Dis.

[7] Cheng VC, Woo PC, Lau SK, Cheung CH, Yung RW, Yam LY (2003). Peripartum tuberculosis as a form of immunorestitution disease. Eur J Clin Microbiol Infect Dis.

[8] Bell LC, Breen R, Miller RF, Noursadeghi M, Lipman M (2015). Paradoxical reactions and immune reconstitution inflammatory syndrome in tuberculosis. Int J Infect Dis.

[9] Bekker LG, Maartens G, Steyn L, Kaplan G (1998). Selective increase in plasma tumor necrosis factor-α and concomitant clinical deterioration after initiating therapy in patients with severe tuberculosis. J Infect Dis.

[10] Kiggundu R, Rhein J, Meya DB, Boulware DR, Bahr NC (2014). Unmasking cryptococcal meningitis immune reconstitution inflammatory syndrome in pregnancy induced by HIV antiretroviral therapy with postpartum paradoxical exacerbation. Med Mycol Case Rep.

[11] Einsiedel L, Gordon DL, Dyer JR (2004). Paradoxical inflammatory reaction during treatment of Cryptococcus neoformans var. gattii meningitis in an HIV-seronegative woman. Clin Infect Dis.

[12] Cheng VC, Ho PL, Lee RA, Chan KS, Chan KK, Woo PC (2002). Clinical spectrum of paradoxical deterioration during antituberculosis therapy in non-HIV-infected patients. Eur J Clin Microbiol Infect Dis.

[13] Breen RA, Smith CJ, Bettinson H, Dart S, Bannister B, Johnson MA (2004). Paradoxical reactions during tuberculosis treatment in patients with and without HIV co-infection. Thorax.

[14] Chakrabarti B, Davies PD (2006). Pleural tuberculosis. Monaldi Arch Chest Dis.

[15] Oge T, Ozalp SS, Yalcin OT, Kabukcuoglu S, Kebapci M, Arik D (2012). Peritoneal tuberculosis mimicking ovarian cancer. Eur J Obstet Gynecol Reprod Biol.

[16] Kasahara K, Fukuoka A, Murakawa K, Okamura H, Mikasa K, Narita N (2005). Tuberculous peritonitis developing during chemotherapy for pulmonary and intestinal tuberculosis: a case report. Respirology.

[17] Lee YJ, Jung SH, Hyun WJ, Kim SH, Lee HI, Yang HW (2009). A case of obstructive jaundice caused by paradoxical reaction during antituberculous chemotherapy for abdominal tuberculosis. Gut Liver.

[18] van den Berge M, de Marie S, Kuipers T, Jansz AR, Bravenboer B (2005). Psoas abscess: report of a series and review of the literature. Neth J Med.

[19] Agarwal R, Suneja A, Raina S. Tuberculous psoas abscess in obstetrics: concerning Kumar S, Malhotra N, Chanana C, Lal S (2009) Psoas abscess in obstetrics. Arch Gynecol Obstet 279:247–249. Arch Gynecol Obstet. 2010;281:575–6. 10.1007/s00404-009-1198-6 19685065

[20] Yamada G, Nishikiori H, Fujii M, Inomata S, Chiba H, Hirokawa N (2013). Systemic lymph node tuberculosis presenting with an aseptic psoas abscess caused by a paradoxical reaction after nine months of antituberculosis treatment: a case report. J Med Case Rep.

